# Comparison of *Crocus sativus L*. and imipramine in the treatment of mild to moderate depression: A pilot double-blind randomized trial [ISRCTN45683816]

**DOI:** 10.1186/1472-6882-4-12

**Published:** 2004-09-02

**Authors:** Shahin Akhondzadeh, Hasan Fallah-Pour, Khosro Afkham, Amir-Hossein Jamshidi, Farahnaz Khalighi-Cigaroudi

**Affiliations:** 1 Psychiatric Research Center, Roozbeh Psychiatric Hospital, Tehran University of Medical Sciences, South Kargar Street, Tehran 13185, Iran; 2 Facuty of Pharmacy, Tehran University of Medical Sciences, Tehran, Iran

## Abstract

**Background:**

The morbidity and mortality associated with depression are considerable and continue to increase. Depression currently ranks fourth among the major causes of disability worldwide, after lower respiratory infections, prenatal conditions, and HIV/AIDS. *Crocus sativus L*. is used to treat depression. Many medicinal plants textbooks refer to this indication whereas there is no evidence-based document. Our objective was to compare the efficacy of stigmas of *Crocus sativus *(saffron) with imipramine in the treatment of mild to moderate depression in a 6-week pilot double-blind randomized trial.

**Methods:**

Thirty adult outpatients who met the Diagnostic and Statistical Manual of Mental Disorders, 4^th ^edition for major depression based on the structured clinical interview for DSM IV participated in the trial. Patients have a baseline Hamilton Rating Scale for Depression score of at least 18. In this double-blind, single-center trial, patients were randomly assigned to receive capsule of saffron 30 mg/day (TDS) (Group 1) and capsule of imipramine 100 mg/day (TDS) (Group 2) for a 6-week study.

**Results:**

Saffron at this dose was found to be effective similar to imipramine in the treatment of mild to moderate depression (F = 2.91, d.f. = 1, P = 0.09). In the imipramine group anticholinergic effects such as dry mouth and also sedation were observed more often that was predictable.

**Conclusion:**

The main overall finding from this study is that saffron may be of therapeutic benefit in the treatment of mild to moderate depression. To the best of our knowledge this is the first clinical trial that supports this indication for saffron. A large-scale trial with placebo control is warranted.

## Background

Depressive disorders are very common in clinical practice, with approximately 11.3% of all adults afflicted during any one year [1]. The majority of patients suffer from mild to moderate forms and are treated in primary care settings. Such patients are often reluctant to take synthetic antidepressants in their appropriate doses due to their anticipated side effects including inability to drive a car, dry mouth, constipation and sexual dysfunction. As a therapeutic alternative, effective herbal drugs may offer advantages in terms of safety and tolerability, possibly also improving patient compliance [2,3]. The advent of the first antidepressants- the Monoamine Oxidase Inhibitors (MAOIs) and Tricyclic Antidepressants (TCAs) in the 1950s and 1960s represented a dramatic leap forward in the clinical management of depression. The subsequent development of the Selective Serotonin Reuptake Inhibitors (SSRIs) and the Serotonin Norepinephrine Reuptake Inhibitor (SNRI) venlafaxine in the past decade and a half has greatly enhanced the treatment of depression by offering patients medications that are as effective as the older agents but are generally more tolerable and safer in an overdose [4,5]. The introduction of atypical antidepressants, such as bupropion, nefazadone, and mirtazapine, has added substantially to the available pharmacopoeia for depression. Nonetheless, rates of remission tend to be low and the risk of relapse and recurrence remains high [2,4]. Thus, there is a need for more effective and less toxic agents. Plants extracts are some of the most attractive sources of new drugs, and have been shown to produce promising results for the treatment of depression [6,7].

Saffron is produced from the tiny, dried stigma of lily-like *Crocus sativus *blossom, genuine saffron is worth its weight in gold. This plant belongs to the Iridaceae family. Although once considered a remedy for digestive problems, saffron is no longer used medicinally in the West [8]. In Asian medicine and in particular Persian traditional medicine, it is used to treat menstrual disorder, difficult labor, inflammation, vomiting, and throat diseases [8-10]. Recent studies indicate its potential as an anti cancer and memory enhancer agent as well [11,12]. Although medicinal plants are used for a wide variety of physical ailments, but often there is limited research supporting these practices. *Crocus sativus *is also used to treat depression [9]. Many Persian medicinal plants textbooks refer to this usage whereas there is no evidence-based document. Our objective was to compare the efficacy of *Crocus sativus *with imipramine in the treatment of mild to moderate depression in a 6-week double blind randomized trial.

## Methods

This was a 6-week randomized and double blind clinical trial. The investigation was conducted in the outpatient clinic of Roozbeh Psychiatric Hospital between January 2002 and February 2004.

### Patients

Thirty adult outpatients who met the Diagnostic and Statistical Manual of Mental Disorders, 4^th ^edition (DSM IV) [13] for major depression based on the structured clinical interview for DSM IV, participated in the trial. Patients have a baseline Hamilton Rating Scale for Depression (HAM-D 17-item) [14] score of at least 18. Prospective participants with the following DSM IV diagnosis were excluded: current cognitive disorder in the last year; or current or past history of bipolar disorder, schizophrenia and schizotypal personality disorder. Patients were required to be free of all psychotropic medications for at least 4 weeks before study entry. Patients were selected to range in age from 18 to 55 years of age. As depression is a serious and potentially life threatening condition and the participants were outpatients so extensive safeguards were needed. Patients were excluded if they posed a significant risk of suicide at any time during participation. Persons who scored greater than 2 on the suicide item of the HAM-D, or who were judged to have significant suicidal ideation or potential in the view of an investigator were excluded. Further, any clinically significant deterioration in the condition of the subject from baseline would result in exclusion. Those who left the study before completion were offered alternative and standard care immediately. Pregnant women or women not using medically accepted means of birth control were excluded. All participants provided written informed consent, and the protocol satisfied the Tehran University of Medical Sciences Ethics Committee requirements.

### Saffron capsule preparation

The saffron was used in this study was dedicated by Novin Zaferan Co (Mashhad, Iran) and was identified by the Department of Cultivation and Development of Institute of Medicinal Plants, Tehran, Iran. The stigma's extract was prepared as follow: 120 g of dried and milled stigmas was extracted with 1800 ml ethanol (80%) by percolation procedure in three steps then the ethanolic extract was dried by evaporation in temperature between 35–40°C. Each capsule had dried extract of saffron (10 mg), lactose (filler), magnesium stearate (lubricant), and sodium starch glycolate (disintegrant).

### Study design

Patients underwent a standard clinical assessment comprising a psychiatric evaluation, a structured diagnostic interview and a medical history. Patients were randomized to receive capsule of saffron or capsule of imipramine in a 1: 1 ratio using a computer-generated code. The investigation preparations were administrated in red capsules whole indistinguishable in color, size, form and taste. The assignments were kept in sealed, opaque envelopes until the point of allocation. The randomization and allocation process was done by the pharmacist of the Roozbeh Hospital. In this double-blind, single-center trial, patients were randomly assigned to receive capsule saffron 30 mg/day (TDS)(Group A) or capsule imipramine 100 mg/day (TDS) for a 6-week study. The dose of saffron was calculated according to a recent published animal study [9]. All patients completed the trial. Patients were assessed by a third year resident of psychiatry at baseline and after 1, 2, 3, 4 and 6 weeks after the medication started. The principal measure of the outcome was the 17-item HAM-D. The rater used standardized instructions in the use of HAM-D. The mean decrease in HAM-D score from baseline was used as the main outcome measure of response of depression to treatment. Throughout the study the person who administrated the medications, rater and patients were blind to assignments.

### Side effects

Side effects were systematically recorded throughout the study and were assessed using a checklist administered by a resident of psychiatry on day 3, 7, 14, 21, 28 and 42 (Table 2).

**Table 2:** Clinical complications and side effects were reported as number per group.

**Side Effects**	**Saffron**	**Imipramine**	**P**
**Anxiety**	4	1	0.32
**Decreased Appetite**	2	0	0.48
**Increased Appetite**	1	5	0.16
**Sedation**	0	6	**0.01**
**Nausea**	2	1	1.00
**Headache**	3	2	1.00
**Dry Mouth**	1	7	**0.03**
**Hypomania**	2	1	1.00
**Constipation**	2	5	038
**Urinary Retention**	1	5	0.16

### Statistical analysis

A two-way repeated measures analysis of variance (time-treatment interaction) was used. The two groups as a between-subjects factor (group) and the six weekly measurements during treatment as the within-subjects factor (time) were considered. This was done for HAM-D total scores. In addition, a one-way repeated measures analysis of variance with a two-tailed post hoc Tukey mean comparison test were performed in the change from baseline for HAM-D scores in each group. To compare the two groups at baseline and the outcome of two groups at the end of the trial, an unpaired Student's t-test with a two-sided P value was used. Results are presented as mean ± S.E.M. Differences were considered significant with P < 0.05. To compare the demographic data and frequency of side effects between the protocols, Fisher's exact test (two sided) was performed. To consider, a = 0.05, β = 0.2, the final difference between the two groups at least score of 5 on the HAM-D total scores that is clinically detectable, S = 5 and power = 80%, the sample size was calculated at least 15 in each group.

## Results

No significant differences were identified between patients randomly assigned to the group 1 or 2 conditions with regard to basic demographic data including age and gender (Table 1).

**Table 1:** Baseline data

	**Saffron Group**	**Imipramine Group**
**Women**	9	8
**Man**	6	7
**Age (Mean ± SD)**	35.53 ± 10.28 (year)	32.53 ± 8.10 (year)
**Baseline Hamilton Score**	19.20 ± 0.42	19.00 ± 0.44

### Efficacy: Saffron versus imipramine

**Figure 1:**
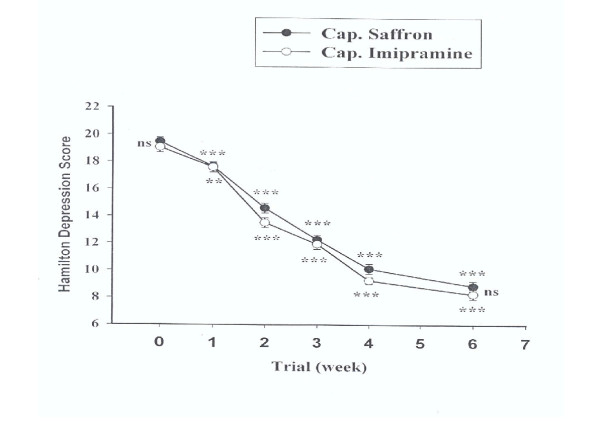
Mean ± SEM scores of two groups of patients on the Hamilton Depression Rating Scale. ns = non-significant, ** = P < 0.01 and *** = P < 0.001. The horizontal symbols (** and ***) were used to express statistical significance versus their respective baseline value and ns symbols are for between group comparisons.

The mean ± SEM scores of groups 1 and 2 are shown in Fig. 1. There were no significant differences between the two groups at week 0 (baseline) on the HAM-D (d.f. = 28, P = 0.43). Both groups showed a significant improvement over the 6 weeks of treatment (P < 0.0001). The difference between the two protocols was not significant as indicated by the effect of group, the between-subjects factor (F = 2.91, d.f. = 1, P = 0.09). The behavior of the two treatment groups was homogeneous across the trial (groups-by-time interaction, F = 0.83, d.f. = 3.44, P = 0.49). In addition, the difference between the two protocols was not significant at week 6 (d.f. = 28, P = 0.33).

### Clinical complications and side-effects

A number of probable side effects were studied (Table 2). Dry mouth and sedation were observed more often in the imipramine group.

## Discussion

The current therapeutic goal in the treatment of major depression is to improve the quality of life by normalizing mood, increasing awareness of personal pleasures and interests, and reversing the functional and social disabilities associated with depression, as well as to reduce suicide rates [1]. Saffron is used in folk medicine as an antispasmodic, eupeptic, gingival, sedative, anticatarrhal, nerve sedative, carminative, diaphoteric, expectorant, stimulant, stomachic, aphrodisiac, antidepressant and emmenagogue [8]. Furthermore, modern pharmacological studies have demonstrated that saffron extract has an anti tumor effect, radical scavenger property and hypolipaemic effect [11]. The present study was carried out to investigate the possible antidepressant effect of saffron compared with imipramine that has already shown to be significantly more efficacious than placebo in the treatment of major depression [2].

In this small preliminary double-blind and randomized comparison of saffron and imipramine in the treatment of mild to moderate depression, saffron at this dose was found to be effective similar to imipramine. Our results are in the line with a recent published animal study that *Crocus sativus *extracts showed antidepressant effect [9]. In addition, in the imipramine group anticholinergic effects such as dry mouth and sedation were observed more often that was predictable. Saffron at this dose did not induce any abnormal bleeding that is one of the reported side effects of it. It has been reported that saffron inhibits platelet adhesion so its use is contraindicated in pregnancy [9]. In addition, it has been suggested that crocin and safranal two major components of saffron inhibit reuptake of dopamine, norepinephrine and serotonin [9]. The limitations of the present study, including lack of a placebo group, using only a fixed dose of saffron, the small number of participants and short period of follow up should be considered so further research in this area is needed. Indeed, patients and their families may view alternative medicine that is, those treatments that are not traditionally taught in medical schools or generally practiced by clinicians, as being complementary or even superior to conventional medicine. In majority of cases there are no evidence-based documents for them. Therefore, the search for new and more effective therapeutic agents includes the scientific study of plants used in traditional medicine systems to treat mental disorders. The main overall finding from this study is that saffron may be of therapeutic benefit in the treatment of mild to moderate depression. A large-scale trial with placebo control is warranted.

## Competing Interest

None declared.

## Authors' contribution

SA was the principal investigator and performed statistical analysis. HF and KA were the trialist. AHJ and FKC were the pharmacognosists of this study. All authors read and approved the final manuscript.

## Pre-publication history

The pre-publication history for this paper can be accessed here:


